# Challenges and disparities in the application of personalized genomic medicine to populations with African ancestry

**DOI:** 10.1038/ncomms12521

**Published:** 2016-10-11

**Authors:** Michael D. Kessler, Laura Yerges-Armstrong, Margaret A. Taub, Amol C. Shetty, Kristin Maloney, Linda Jo Bone Jeng, Ingo Ruczinski, Albert M. Levin, L. Keoki Williams, Terri H. Beaty, Rasika A. Mathias, Kathleen C. Barnes, Consortium on Asthma among African-ancestry Populations in the Americas (CAAPA), Meher Preethi Boorgula, Monica Campbell, Sameer Chavan, Jean G. Ford, Cassandra Foster, Li Gao, Nadia N. Hansel, Edward Horowitz, Lili Huang, Romina Ortiz, Joseph Potee, Nicholas Rafaels, Alan F. Scott, Candelaria Vergara, Jingjing Gao, Yijuan Hu, Henry Richard Johnston, Zhaohui S. Qin, Badri Padhukasahasram, Georgia M. Dunston, Mezbah U. Faruque, Eimear E. Kenny, Kimberly Gietzen, Mark Hansen, Rob Genuario, Dave Bullis, Cindy Lawley, Aniket Deshpande, Wendy E. Grus, Devin P. Locke, Marilyn G. Foreman, Pedro C. Avila, Leslie Grammer, Kwang-YounA Kim, Rajesh Kumar, Robert Schleimer, Carlos Bustamante, Francisco M. De La Vega, Chris R. Gignoux, Suyash S. Shringarpure, Shaila Musharoff, Genevieve Wojcik, Esteban G. Burchard, Celeste Eng, Pierre-Antoine Gourraud, Ryan D. Hernandez, Antoine Lizee, Maria Pino-Yanes, Dara G. Torgerson, Zachary A. Szpiech, Raul Torres, Dan L. Nicolae, Carole Ober, Christopher O. Olopade, Olufunmilayo Olopade, Oluwafemi Oluwole, Ganiyu Arinola, Wei Song, Goncalo Abecasis, Adolfo Correa, Solomon Musani, James G. Wilson, Leslie A. Lange, Joshua Akey, Michael Bamshad, Jessica Chong, Wenqing Fu, Deborah Nickerson, Alexander Reiner, Tina Hartert, Lorraine B. Ware, Eugene Bleecker, Deborah Meyers, Victor E. Ortega, Maul R. N. Pissamai, Maul R. N. Trevor, Harold Watson, Maria Ilma Araujo, Ricardo Riccio Oliveira, Luis Caraballo, Javier Marrugo, Beatriz Martinez, Catherine Meza, Gerardo Ayestas, Edwin Francisco Herrera-Paz, Pamela Landaverde-Torres, Said Omar Leiva Erazo, Rosella Martinez, Alvaro Mayorga, Luis F. Mayorga, Delmy-Aracely Mejia-Mejia, Hector Ramos, Allan Saenz, Gloria Varela, Olga Marina Vasquez, Trevor Ferguson, Jennifer Knight-Madden, Maureen Samms-Vaughan, Rainford J. Wilks, Akim Adegnika, Ulysse Ateba-Ngoa, Maria Yazdanbakhsh, Timothy D. O'Connor

**Affiliations:** 1 Institute for Genome Sciences, University of Maryland School of Medicine, Baltimore, Maryland 21201, USA; 2 Department of Medicine, University of Maryland School of Medicine, Baltimore, Maryland 21201, USA; 3 Program in Personalized and Genomic Medicine, University of Maryland School of Medicine, Baltimore, Maryland 21201, USA; 4 Department of Biostatistics, Bloomberg School of Public Health, Johns Hopkins University, Baltimore, Maryland 21287, USA; 5 Department of Public Health Sciences, Henry Ford Health System, Detroit, Michigan 48202, USA; 6 Center for Health Policy & Health Services Research, Henry Ford Health System, Detroit, Michigan 48202, USA; 7 Department of Internal Medicine, Henry Ford Health System, Detroit, Michigan 48202, USA; 8 Department of Epidemiology, Bloomberg School of Public Health, Johns Hopkins University, Baltimore, Maryland 21205, USA; 9 Department of Medicine, Johns Hopkins University, Baltimore, Maryland 21224, USA; 10 Department of Medicine, University of Colorado, Aurora, Colorado 80045, USA; 11 Department of Medicine, The Brooklyn Hospital Center, Brooklyn, New York, USA; 12 Data and Statistical Sciences, AbbVie, North Chicago, Illinois, USA; 13 Department of Biostatistics and Bioinformatics, Emory University, Atlanta, Georgia, USA; 14 Department of Microbiology, Howard University College of Medicine, Washington, DC, USA; 15 National Human Genome Center, Howard University College of Medicine, Washington, DC, USA; 16 Department of Genetics, Stanford University School of Medicine, Stanford, California, USA; 17 Department of Genetics and Genomics, Icahn School of Medicine at Mount Sinai, New York, New York, USA; 18 Illumina, Inc., San Diego, California, USA; 19 Knome Inc., Cambridge, Massachusetts, USA; 20 Pulmonary and Critical Care Medicine, Morehouse School of Medicine, Atlanta, Georgia, USA; 21 Department of Medicine, Northwestern University, Chicago, Illinois, USA; 22 Department of Preventive Medicine, Northwestern University, Chicago, Illinois, USA; 23 Department of Pediatrics, Northwestern University, Chicago, Illinois, USA; 24 The Ann & Robert H. Lurie Children's Hospital of Chicago, Chicago, Illinois, USA; 25 Department of Medicine, Northwestern Feinberg School of Medicine, Chicago, Illinois, USA; 26 Department of Bioengineering and Therapeutic Sciences, University of California, San Francisco, San Francisco, California, USA; 27 Department of Medicine, University of California, San Francisco, San Francisco, California, USA; 28 Department of Neurology, University of California, San Francisco, San Francisco, California, USA; 29 Institute for Human Genetics, University of California, San Francisco, San Francisco, California, USA; 30 California Institute for Quantitative Biosciences, University of California, San Francisco, San Francisco, California, USA; 31 CIBER de Enfermedades Respiratorias, Instituto de Salud Carlos III, Madrid, Spain; 32 Biomedical Sciences Graduate Program, University of California, San Francisco, San Francisco, California, USA; 33 Department of Medicine, University of Chicago, Chicago, Illinois, USA; 34 Department of Statistics, University of Chicago, Chicago, Illinois, USA; 35 Department of Human Genetics, University of Chicago, Chicago, Illinois, USA; 36 Department of Medicine and Center for Global Health, University of Chicago, Chicago, Illinois, USA; 37 Department of Chemical Pathology, University of Ibadan, Ibadan, Nigeria; 38 Department of Biostatistics, SPH II, University of Michigan, Ann Arbor, Michigan, USA; 39 Department of Medicine, University of Mississippi Medical Center, Jackson, Mississippi, USA; 40 Department of Physiology and Biophysics, University of Mississippi Medical Center, Jackson, Mississippi, USA; 41 Department of Genetics, University of North Carolina, Chapel Hill, North Carolina, USA; 42 Department of Genomic Sciences, University of Washington, Seattle, Washington, USA; 43 Department of Pediatrics, University of Washington, Seattle, Washington, USA; 44 University of Washington, Seattle, Washington, USA; 45 Department of Medicine, Vanderbilt University, Nashville, Tennessee, USA; 46 Department of Pathology, Microbiology and Immunology, Vanderbilt University, Nashville, Tennessee, USA; 47 Center for Human Genomics and Personalized Medicine, Wake Forest School of Medicine, Winston-Salem, North Carolina, USA; 48 Genetics and Epidemiology of Asthma in Barbados, The University of the West Indies, West Indies; 49 Faculty of Medical Sciences Cave Hill Campus, The University of the West Indies, West Indies; 50 Queen Elizabeth Hospital, The University of the West Indies, West Indies; 51 Immunology Service, Universidade Federal da Bahia, Salvador, Brazil; 52 Laboratório de Patologia Experimental, Centro de Pesquisas Gonçalo Moniz, Salvador, Brazil; 53 Institute for Immunological Research, Universidad de Cartagena, Cartagena, Spain; 54 Instituto de Investigaciones Immunologicas, Universidad de Cartagena, Cartagena, Spain; 55 Faculty of Medicine, Universidad Nacional Autonoma de Honduras en el Valle de Sula, San Pedro Sula, Honduras; 56 Facultad de Medicina, Universidad Catolica de Honduras, San Pedro Sula, Honduras; 57 Centro de Neumologia y Alergias, San Pedro Sula, Honduras; 58 Faculty of Medicine, Centro Medico de la Familia, San Pedro Sula, Honduras; 59 Tropical Medicine Research Institute, The University of the West Indies, West Indies; 60 Department of Child Health, The University of the West Indies, West Indies; 61 Centre de Recherches Médicales de Lambaréné, Libreville, Gabon; 62 Institut für Tropenmedizin, Universität Tübingen, Tübingen, Germany; 63 Department of Parasitology, Leiden University Medical Center, Leiden, Netherlands; 64 A full list of consortium members appears at the end of the paper.

## Abstract

To characterize the extent and impact of ancestry-related biases in precision genomic medicine, we use 642 whole-genome sequences from the Consortium on Asthma among African-ancestry Populations in the Americas (CAAPA) project to evaluate typical filters and databases. We find significant correlations between estimated African ancestry proportions and the number of variants per individual in all variant classification sets but one. The source of these correlations is highlighted in more detail by looking at the interaction between filtering criteria and the ClinVar and Human Gene Mutation databases. ClinVar's correlation, representing African ancestry-related bias, has changed over time amidst monthly updates, with the most extreme switch happening between March and April of 2014 (*r*=0.733 to *r*=−0.683). We identify 68 SNPs as the major drivers of this change in correlation. As long as ancestry-related bias when using these clinical databases is minimally recognized, the genetics community will face challenges with implementation, interpretation and cost-effectiveness when treating minority populations.

## Abstract (web summary)

Personalized medicine requires accurate and ethnicity-optimized reference genome panels. Here, the Consortium on Asthma among African-ancestry Populations in the Americas (CAAPA) evaluates typical variant filters and existing genome databases against newly sequenced African-ancestry populations.

## Graphical abstract

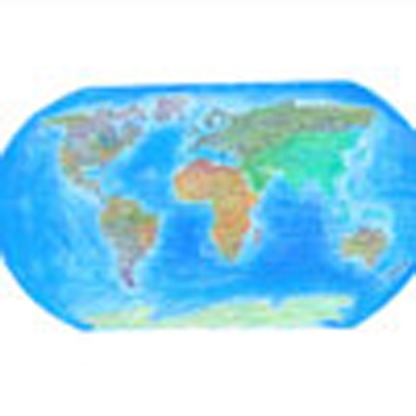

The idiom ‘searching for a needle in a haystack' is frequently used in genomics, and is especially apt for describing the search for causal alleles in patients with non-canonical diseases of likely genetic origin. As a field, we tend to be singularly focused on the needle and forget that the complexity of the haystack is actually a highly rate-limiting step of this search. The motivation of this project is to characterize the complex interaction between variant prioritization and ancestry, often believed to be largely affected by the predominance of European-based data within clinical databases^1^,^2^,^3^, to better understand the application of clinical genomics to minority populations. Any ancestry-related biases that exist when using typical filters and databases to implement variant prioritization and other similar precision genomic medicine techniques can have profound confounding effects, as most methodological biases do. Therefore, here we quantify the extent of ancestry-related biases inherent to approaches and databases typically used for precision genomic medicine, and we present how such biases have changed over time. We also show how these biases translate to the level of the individual and their proportion of African ancestry, with implications for diagnostic accuracy and cost.

To explore the role ancestry plays in variant prioritization approaches often implemented in genomic medicine, we utilize whole-genome sequencing data from 642 study individuals in the Consortium on Asthma among African-ancestry Populations in the Americas (CAAPA). The CAAPA project represents a diverse group of admixed individuals of African descent with no suspected Mendelian conditions. It has been shown that there is a strong correlation between the overall number of variants found per individual and African ancestry^4^,^5^,^6^,^7^. Furthermore, significant differences exist between populations in the number of variants per individual considered disease causing by the two popular clinical databases, Human Gene Mutation Database (HGMD) and ClinVar^6^. On the basis of annotations from HGMD, individuals with predominantly African ancestry have by far the most variants considered disease causing, whereas variants prioritized as disease causing based on annotations from ClinVar are most abundant in individuals with predominantly European ancestry and are of intermediate to below-average abundance in predominantly African-ancestry individuals^6^. These population-based discrepancies reflect differences between databases, and suggest that the interplay between database and sample ancestry is important. The CAAPA cohort utilized here serves as an appropriate sample, with representative quantities of variation (that is, similar-sized haystacks), for evaluating whether biases exist when applying precision genomic medicine to African-ancestry individuals. Any biases and/or population specificities for African-ancestry patients that inflate the number of prioritized variants (that is, make the haystack bigger), would result in increased effort (that is, time and money) to identify a causative variant (that is, find the needle) in African-ancestry patients.

## Results

### Variant classification

**Figure 1:**
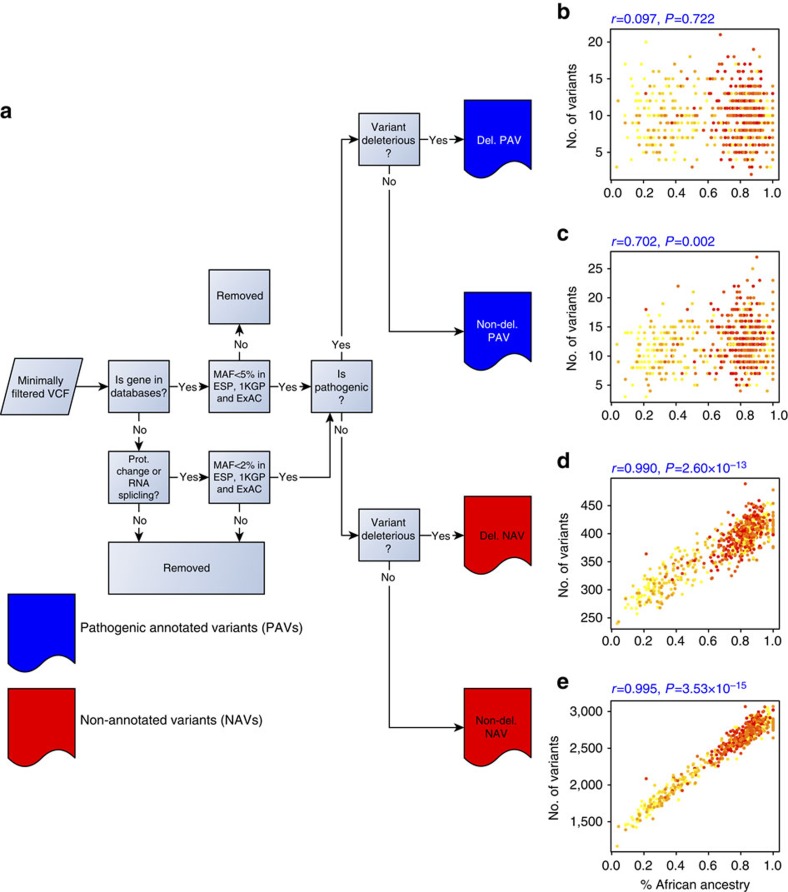
Variant classification workflow and correlation with African ancestry. (**a**) The pipeline used to categorize variation into four groups (deleterious (Del.) PAVs, non-deleterious (Non-del.) PAVs, deleterious NAVs and non-deleterious NAVs) each with different levels of clinical relevance (see Methods for further explanation). The three key filters used in separating variants are (1) MAF from multiple databases, (2) pathogenic annotation (as defined by the ClinVar and/or HGMD) and (3) deleterious prediction. For **b**–**e** the *x* axis is the proportion of African ancestry as estimated by ADMIXTURE. The corresponding *y* axes represent the total number of variants per individual for the following groups: (**b**) deleterious PAVs, (**c**) non-deleterious PAVs, (**d**) deleterious NAVs and (**e**) non-deleterious NAVs. Colours of each individual reflect the population sampling location.

We initially classified variants into two main groups, with pathogenic annotated variants (PAVs) comprising those identified as disease-causing in the Online Mendelian Inheritance in Man (OMIM)^8^, HGMD^9^ or ClinVar^10^ databases, and non-annotated variants (NAVs) consisting of those not annotated as disease-causing in these databases. Unless otherwise noted, we used a an allele frequency filter, and excluded common variants with a minor allele frequency (MAF) >5% from our analyses (Methods). Each category was then sub-classified as deleterious or non-deleterious based on computational predictions (Fig. 1a)^11^,^12^,^13^,^14^,^15^,^16^,^17^,^18^,^19^,^20^, which we consider as a type of filter based on deleteriousness and note when used for categorization. Since there is evidence for all PAVs (deleterious and non-deleterious) and deleterious NAVs to be further evaluated as higher priority, variants in these categories often require time-consuming and costly follow-up review by a clinical team^1^,^21^,^22^ to identify causative variants with a low false-negative rate.

### Correlations with African ancestry and variant counts

We find significant correlations between estimated African ancestry (Supplementary Fig. 1) and the number of variants per individual in all variant sets except deleterious PAVs (Fig. 1b–d). Both deleterious and non-deleterious NAVs show similar levels of correlation with African ancestry as does all genomic variation pooled together^7^. When we remove the aforementioned MAF and deleteriousness filters, as well as a filter on stop/splice sites, and identify PAVs from either HGMD or ClinVar databases separately, we find a strong positive correlation between estimated African ancestry and variants identified in HGMD (*r*=0.992, *P*=6.12 × 10^−14^) and a modest positive correlation between African ancestry and variants in ClinVar (*r*=0.539, *P*=0.031). The correlation becomes less positive or even negative (Supplementary Table 1) as we re-add our two main filters: (1) inclusion of variants with MAF <5% (MAF filter); and (2) inclusion of variants called deleterious by at least 2 of 11 *in silico* predictions (deleterious filter).

One possible explanation for this general reduction of the positive correlation with ancestry is that these filters effectively remove functionally neutral variants, of which there are more in persons of African ancestry. Assuming this, one would predict a reduction in the positive correlation with African ancestry, as long as the filters remove a higher number of functionally neutral variants, relative to causative variants, from African populations compared with European populations. Given recent studies showing that African populations have more genetic variation than European populations^5^,^23^,^24^, but that the number of deleterious alleles in an individual is independent of demography or lower in Africans, depending on the level of deleteriousness of these alleles^25^,^26^,^27^,^28^,^29^, one would expect all filters to remove higher numbers of non-causal variants from individuals with greater African ancestry, as is consistent with what we report here. Specifically, as we apply the MAF filter and exclude all common variants, we are eliminating variants that have been misidentified in databases as disease causing^22^, of which there are more among individuals of African ancestry. Similarly, as we use *in silico* predictors to filter out putatively non-deleterious variants, we remove more functionally neutral variants from Africans than from Europeans. For instance, the number of non-deleterious PAVs per individual increases with African ancestry, whereas the number of deleterious PAVs per individual does not. Furthermore, because the number of deleterious mutations in African individuals is not greater than in European individuals^25^,^26^,^27^,^28^, these filters do not remove more deleterious variants from Africans. This disproportionate removal of functionally neutral variants will more effectively reduce the number of incorrectly characterized variants in each class in African-ancestry individuals, and explains the reduction of the positive correlation with African ancestry as filters are applied.

### Deleterious predictors are different depending on annotation

While filtering significantly reduces the correlation between the number of deleterious PAVs and African ancestry, it does not impact the correlation between the number of deleterious NAVs and African ancestry. One possible explanation is that the effects of the filters differ between the two categories of variants, with functionally neutral variation filtered out more efficiently for PAVs. Because we require at least 2 of 11 predictors to call a variant putatively deleterious, it is possible that predictors calling PAVs deleterious are consistently different than those calling NAVs deleterious. This is what we observe (*P*≤10^−15^, *χ*^2^-test of independence), with predictors that use clinical databases to train their algorithms being over represented in deleterious PAV calls, and algorithms that are agnostic to clinical databases making up a larger percentage of the deleterious NAV calls (Supplementary Table 5). One possibility is that the machine learning-based algorithms preferentially optimize for patterns within the PAVs, and can thus inherit an ancestry-specific bias. Supporting this is the notion that most new African-specific causal variants will initially be identified as NAVs and may thus be less likely to be called by the currently trained predictors. Alternatively, though not mutually exclusively, conservation algorithms may be better able to remove background variants from the NAVs if the conservation score range for NAVs is significantly larger than PAVs. Given that NAVs are not annotated and are less processed than PAVs, they are more likely to be sampled equally across the entire distribution of conservation scores, and to therefore represent a wider range of conservation scores than PAVs. This is consistent with what others have observed^2^, and might explain why conservation algorithms predominate in the separation of deleterious NAVs from non-deleterious NAVs, compared with PAVs. While this differences in the type of predictors used in distinguishing deleterious and non-deleterious variants of different classification may represent the potential extension of ancestry related biases to deleterious predictors, this needs to be studied in more detail.

### ClinVar correlation with African ancestry over time

**Figure 2:**
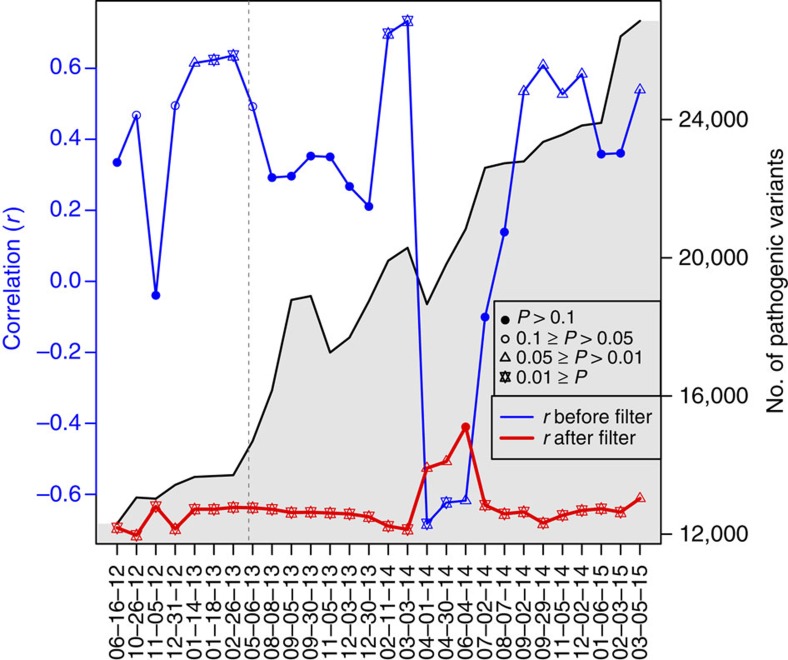
Historical view of African-ancestry biases in ClinVar. The *x* axis represents the various archived versions of ClinVar. In the black *y* axis on the right, we see the number of PAVs recorded from each version of the database. There are a few decreases in numbers, but overall this number shows continuous growth. In the blue *y* axis on the left, we see the correlation coefficient estimated between the number of PAVs per CAAPA individual and their proportion of African ancestry. The dotted grey line represents the date of the first official release of ClinVar. The blue trend line shows the instability across different ClinVar releases of the correlation of African-ancestry proportion with average number of pathogenic variants per individual. The change in correlation is particularly notable for sequential releases between March and April 2014, after which the correlation remains significantly negative for 3 months (April–July 2014) before once again becoming significantly positive. The red trend line represents the same relationship between ancestry–pathogenicity correlation and ClinVar release over time after applying filters, and shows a significant change in correlation during the same 3-month period of April–July 2014 despite an overall reduction in movement of the correlation across time.

To explore the historical context of recognized PAVs, and evaluate how ancestry related biases may have impacted the reproducibility of previous clinical applications relying on ClinVar, we conducted an analysis of how biases in archived versions of ClinVar have changed over time. ClinVar, a developing database of pathogenic variation officially released in April 2013, was chosen for this analysis as it has monthly updates that allow us to easily track changes over time. As of March 2015, the number of known pathogenic variants has almost doubled from 14,697 to 26,409. In Fig. 2, we show the correlation over time between African-ancestry proportion in our CAAPA individuals and counts of ClinVar-based pathogenic variants in these same individuals for each update between 16 June 2012 (pre-official release) and 5 March 2015. As seen from this figure, the content of the database is highly susceptible to ancestry-related biases, which affects the interpretation of results. Furthermore, these biases can change over time, further complicating the ability to interpret results and account for ancestry-related biases. The largest change happens over a single month, from March to April of 2014, when a significant positive correlation (*r*=0.733, *P*=0.001) switches to a significant negative correlation (*r*=−0.683, *P*=0.004). An analysis of differences between the March and April 2014 releases identifies 68 single-nucleotide polymorphisms (SNPs) that drive this marked change, and more details are presented in the supplement (Supplementary Note 1 and Supplementary Table 3).

The red line near the bottom of Fig. 2 shows the same correlation over time after filtering the data, again by MAF, mutation type and deleterious predictions (Fig. 1a). Similar to the unfiltered data, the filtered data show the first major shift in correlation from March to April of 2014, but the shift is in the opposite direction, with April showing a significantly less negative correlation (stats test) compared with March. The filtered data continue to show a less negative correlation for 3 months, before the pattern returns to a more significant negative correlation in July 2014, which is again similar in trend to but opposite in direction from the pattern seen in the unfiltered analysis. These simultaneous similarities and differences in the shift of the correlation between ancestry and pathogenic variation across database releases and filtering procedures reflect the precariousness of the current clinical databases, particularly when prioritizing variants of individuals with significant non-predominantly European ancestry. In contacting ClinVar about any possible curation differences for the March to July 2014 releases, we learned that ClinVar received a large deposit in April 2014 from the Breast Cancer Information Core database^30^ with significant amounts of non-European data. While further information about this deposit is unavailable and exactly why it caused a marked change from positive to negative correlation is currently unclear, these observations further support our message that database content reflects ancestry-related biases and can impact overall reproducibility.

### Analysis of ancestral biases at the gene level

To explore ancestral biases at a gene level, we evaluated the correlation between the number of PAVs per gene and African ancestry using the March 2015 release of ClinVar. After correcting for multiple testing, we found a significant negative correlation with African ancestry for 10 genes (Supplementary Table 2). These genes represent a subset with the strongest bias, and while we suspect this negative correlation with African ancestry is likely due to some type of technical or ascertainment bias, it is nevertheless possible that this bias could have some biological basis. These genes will require particular care in clinical analysis and represent an interesting set for follow-up investigation, as African causal variants in these genes are more likely to be labelled as NAVs and require a greater identification effort. We find, in general, that the subset of genes with significant positive or negative correlations (*P*<0.05, uncorrected) are not enriched for those genes associated with known Mendelian diseases or those found in the GWAS catalogue^31^ (Methods).

## Discussion

The ability to accurately report whether a genetic variant is responsible for a given disease or phenotypic trait depends in part on the confidence in labelling a variant as pathogenic. Such determination can often be more difficult in persons of predominantly non-European ancestry, as there is less known about the pathogenicity of variants that are absent from or less frequent in European populations. A key part of this are the differences between pathogenic variants, deleterious variants and prioritized variants, which are merely members of the proverbial haystack with differing levels of evidence for potential disease causality. It is important to note that a deleterious variant will only be labelled as pathogenic if its effect size is large enough to directly cause disease and this effect has been seen and annotated, and that a pathogenic variant will only be deleterious if it negatively impacts reproductive fitness. These terms are not the same, nor synonyms of true causality, but the use of deleteriousness as evidence for true disease causality is predicated on the fact that deleteriousness and pathogenicity should be correlated. While we cannot be sure which of these variants are truly disease-causing (actual ‘needles' rather than haystack members) without additional functional or association-based evidence, we believe that discrepancies between true pathogenicity and annotated pathogenicity are a major source of the biases we report. A likely contributor to this incongruity is that databases are missing population-specific pathogenicity information, and with regard to the results we report here, African-specific pathogenicity data. Therefore, true causal variants for predominantly non-European patients are likely to fall into the NAV categories. Since NAVs have the highest degree of positive correlation with African ancestry (that is, bias), causal variants falling into this group are more difficult to distinguish, as they exist amongst a larger number of high-priority background variants (that is, larger haystack). This problem is compounded in individuals of substantial African ancestry, as their larger amount of overall genetic variation^5^,^23^,^24^ results in an even greater number of deleterious NAVs requiring adjudication.

As a consequence, review of genomic test results for persons of predominantly non-European ancestry could be both more challenging and costly. Positive correlations of African-ancestry proportion with non-deleterious PAVs and deleterious NAVs result in more variants to evaluate for African-ancestry individuals (that is, larger haystack), which leads to higher costs and longer turnaround times. Assuming a cost of $500 per variant for Sanger confirmation in a CLIA-certified laboratory (see Supplementary Table 6 for the range of costs found in clinical laboratories), and given gene candidate prioritization approaches that use phenotype to gene mapping^32^ and limit variants receiving follow-up confirmation to those in about 1% of the genome (that is, about 200 genes), we estimate an African-ancestry patient would have about 4.5 prioritized variants needing validation compared with 2.8 in an individual of European ancestry. This translates to a 1.6-fold increase in the number of variants prioritized, and represents a confirmation cost difference of over $800 per patient. Notably, these estimates are simplified and conservative, as we do not consider the substantial cost of having each of these variants reviewed by a clinician.

A potential solution would be to reserve follow-up confirmation for deleterious PAVs, which are uncorrelated to African ancestry and should therefore not be more common in individuals of African ancestry. However, doing this would limit the diagnostic landscape for both Europeans and non-Europeans to only previously found variation, and would greatly undermine the promise that sequencing technology holds for clinical genomics. Furthermore, this would limit the field to Euro-centric databases that would frequently miss causal variants in minority populations. In these situations, the missed causal variants would only be represented among the NAVs, which underlines the importance of not excluding prioritized NAVs from follow-up analysis.

These limitations translate into serious challenges, and despite the increased costs, provide good reason to cast a wider net for variant prioritization and confirmation when applying genomic testing to patients of African ancestry, and likely other predominantly non-European ancestries. As long as ancestry-related biases are not addressed, and most studies continue to predominantly sample from European populations, the genetics community will face challenges with implementation, interpretation and cost-effectiveness when treating minority populations.

## Methods

### Filtering pipeline

We annotate all variation using ANNOVAR^33^, a programme that facilitates the comprehensive and integrative annotation of multiple data types for each variant. Variants are divided into two main classes, each with two subgroups, for a total of four categories. PAVs consist of variants annotated as pathogenic in clinically annotated genetic databases, and are subdivided into deleterious and non-deleterious subgroups as determined by *in silico* predictions. NAVs include all variants not annotated as pathogenic (labelled as disease mutations), as well as those entirely absent from clinically annotated databases, and are also subdivided into deleterious and non-deleterious subgroups. Using customized ANNOVAR index tables, we annotate variants with 11 *in silico* predictors of function^11^,^12^,^13^,^14^,^15^,^16^,^17^,^18^,^19^,^20^ (Supplementary Table 4), functional information about protein-coding effect, clinical variation knowledge from ClinVar^10^ (all archived versions from 2012 to March 2015), the professional version of HGMD^9^ (fourth quarter version of 2014) and allele frequencies from multiple population sequencing projects, including the 1000 Genomes Project (phase 3)^23^, the ExAC database (http://exac.broadinstitute.org) and the Exome Sequencing Project^5^. We also integrate the final output as a list of variants belonging to each of the variant classes described above (Fig. 1a)^1^.

### Filtering criteria

For variants from the OMIM^8^, HGMD^9^ and ClinVar^10^ databases, only those found in protein-coding genes are included. We also remove variants with MAF >5% in any of the 1000 Genome super-populations, ExAC populations or Exome Sequencing Project populations. With regard to the analysis portrayed in Fig. 1, if a variant is not found in any of the clinical databases, we use an allele frequency cutoff of 2% (Fig. 1a) and include only protein-altering variants found in the three following gene annotation databases: ENSEMBL GENE; KnownGene; or RefSeq. We also filter variants on the basis of *in silico* prediction, and require that at least 2 of 11 *in silico* prediction methods identify variants as deleterious (see Supplementary Table 4 for individual predictor cutoffs). An exception to this is that nonsense and splice site variants are called deleterious irrespective of their *in silico* predictors. Situations where these predicted deleteriousness filters are not applied are identified as exceptions in the text.

### Variant classes

The first variant class, deleterious PAVs, are defined as variants with exact matches in genes in the OMIM^8^, HGMD^9^ or ClinVar^10^ databases, and are known to be associated with disease phenotypes. In addition, this class has to meet the above *in silico* prediction filter. The second class of variants is non-deleterious PAVs, and they only differs from the first category in that the requirement of being deleterious is removed. Deleterious NAVs make up the third class. This class is not annotated as pathogenic in any of the clinical databases, but these variants are identified by at least two *in silico* predictors as being deleterious. Finally, variants neither previously annotated as pathogenic nor predicted to be deleterious by at least two *in silico* predictors are classified as non-deleterious NAVs; they are seen as the least likely to be causative for a known disorder. Non-deleterious NAVs are also filtered by the frequency filters described above. Overall, <1% of NAVs are found in databases but not annotated as disease-causing; the remaining NAVs are not identified in any database.

### Whole-genome sequencing data from the CAAPA Project

CAAPA consists of high coverage (∼30 × ) whole-genome sequence data (*N*=642) and provides a catalogue of genetic diversity from multiple populations of African descent. These populations include individuals from North America, South America, the Caribbean and continental Africa^7^, and study individuals are categorized as being cases and controls for asthma (sampling and variant calling are presented in more detail in Mathias *et al*.^7^) However, we do not suspect an atypical number of clinically relevant pathogenic variants among cases with such a complex disease phenotype as asthma. We have sampled 16 populations, including 8 different African American populations. Assembly of individual genomes, as well as variant calls are done using the Consensus Assessment of Sequence and Variation (CASAVA) package^34^. Using probabilistic models to build probability distributions over all diploid genotypes at every genomic site, genotypes are called after numerous quality control filtering steps. For each genomic position, a set of candidate SNPs becomes output. Multi-sample VCFs are generated at Knome Inc. (Cambridge, MA, USA), using VCFtools v0.1.11 (ref. 35) and custom scripts for additional data processing. While everything we report is based on a genome sequencing data set containing 642 samples, when we repeated our analyses on an expanded set of samples (total *N*=∼950) containing additional currently unpublished CAAPA data, our results were unchanged.

### Estimation of ancestry proportions

To estimate ancestry we combine the CAAPA data with phase 1 of the 1000 Genomes Project, and data from previously published studies, which genotyped Hispanic and Native American samples on an Affymetric 6.0 chip^36^,^37^. All A–T and G–C SNPs are removed, and a missingness filter of 5% and a MAF filter of <5% are applied. The resulting SNPs are then LD pruned with plink^38^ using windows of 50 SNPs and removing SNPs with an *r*^2^>0.25, then iterating by 5 SNPs (that is, plink command—indep-pairwise 50 5 0.25). This results in 167,987 SNPs for admixture analysis.

We estimate ancestry proportions using the software package ADMIXTURE^39^. After performing 30 replicates modelling four clusters, we select the parameter values with the highest negative log likelihood. We identify the cluster that represents African ancestry by using the African groups from the 1000 Genomes Project as a reference (that is, the cluster where they have >99% membership), and we extract the proportion estimates for each of our CAAPA samples from this cluster. These become the values used to estimate the correlations. We present them as a bar plot in Supplementary Fig. 1.

### Statistics to accommodate sampling structure

Owing to our population sampling approach, the full cohort does not represent an unstructured selection of individuals of African ancestry. To account for this when performing correlation analysis, we use the approach implemented in the R package ‘psych'^40^. The approach estimates correlations within each single population, which represent the pillars of the population substructure, and then combines these estimates weighted by sample size. Reported correlation coefficients and the *P* values are from the ‘weights'^41^ package, and significance is reported with a false discovery rate approach to correct for multiple testing.

### Data availability

The whole-genome sequence data referenced in this study were generated by the CAAPA^7^ and have been deposited in dbGAP with the accession code phs001123.v1.p1.

## Additional information

**How to cite this article:** Kessler, M. D. *et al*. Challenges and disparities in the application of personalized genomic medicine to populations with African ancestry. *Nat. Commun.* 7:12521 doi: 10.1038/ncomms12521 (2016).

## Supplementary Material

Supplementary Information Supplementary Figure 1, Supplementary Tables 1-6, Supplementary Note 1, Supplementary Discussion, Supplementary Methods and Supplementary References
