# Supplementary material for: Challenges and disparities in the application of personalized genomic medicine to populations with African ancestry
Source: Nat Commun. 2016 Oct 11;7:12521. doi: 10.1038/ncomms12521 (PMC5062569; doi:10.1038/ncomms12521)
Supplement: Supplementary Information — Supplementary Figure 1, Supplementary Tables 1-6, Supplementary Note 1, Supplementary Discussion, Supplementary Methods and Supplementary References [file ncomms12521-s1.pdf]

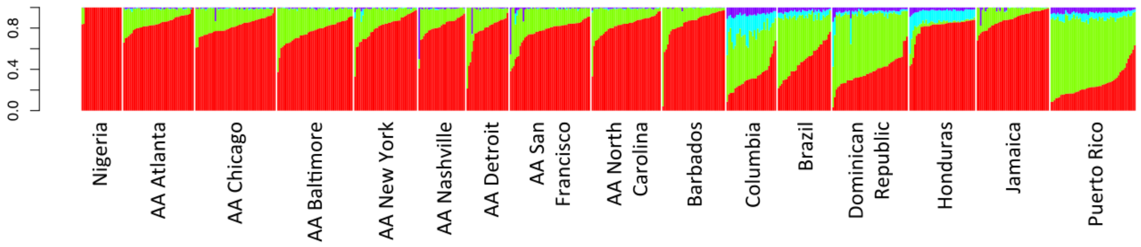


Supplementary Figure 1. Admixture plot of 642 CAAPA samples. This is the admixture estimation results, which also included non-admixted populations from phase 1 of 1000 Genomes Project and the Native Americans from Bigham et al. 2010 as mentioned in the main text in the section titled “Estimation of Ancestry Proportions.” The proportion of African ancestry (red) was used as a key correlate to the variation we found for different categories.

Supplementary Table 1. Correlation of ancestry with number of PAVs per individual identified separately from each of the two databases with and without filtering. Full filtering implies the allele frequency filter and either a deleterious filter or Stop/Splice site. Correlation values are shown for PAVs found in only ClinVar, only HGMD, and either one. For each of these categories, correlation values are presented before filtering, after filtering out variants with a MAF > 0.05 in any of a number of populations, after filtering variants called deleterious by at least two *in silico* predictors, including stop or splice sites, and after all of these filters. Regardless of database origin, each time a filter is added, the positive correlation is reduced. With both filters added, PAVs from ClinVar show a significant negative correlation, while PAVs from HGMD or the union of ClinVar and HGMD show no correlation.

| **Databases** | **No Filtering** | **Deleterious filter** | **AF ≤ 0.05 filter** | **Stop/Splice sites** | **Full filter** |
|---|---|---|---|---|---|
| ClinVar | r=0.539 p=0.031 | r=-0.644 p=0.007 | r=-0.549 p=0.028 | r=0.249 p=0.352 | r=-0.612 p=0.012 |
| HGMD | r=0.992 p=6.12x10-14 | r=0.889 p=3.99x10-6 | r=0.703 p=0.002 | r=-0.183 p=0.498 | r=0.344 p=0.192 |
| HGMD and ClinVar | r=0.989 p=4.82x10-13 | r=0.554 p=0.026 | r=0.551 p=0.027 | r=0.094 p= 0.730 | r=0.094 p=0.729 |

Supplementary Table 2. Genes significantly correlated with African Ancestry. Genes whose pathogenic annotated variants (PAVs) were significantly correlated with African Ancestry are listed. No genes had statistically significant positive correlation with African Ancestry. In other words, these correlations were negative and so individuals with greater African ancestry had fewer pathogenic variants in these genes. Significance was calculated after correcting for multiple testing (Bonferonni correction): Two asterisks (**) signify family-wide significance at the 0.05 level before removing genes with a minimum number of total pathogenic variants summed across all individuals, and a single asterisk (*) signifies similar significance after removal of such genes (representing increased power via removing weak signal genes and reducing number of statistical tests).

| **Gene Symbol** | **r** | **P-Value** |
|---|---|---|
| AXIN1 | -0.778 | 5.44e-05* |
| CHD1L | -0.783 | 4.40e-05* |
| POMGNT1 | -0.794 | 2.87e-05* |
| ORC4 | -0.799 | 2.41e-05* |
| PKP2 | -0.822 | 8.49e-06* |
| NAT1 | -0.836 | 4.42e-06* |
| INO80 | -0.842 | 3.18e-06* |
| BMPR2 | -0.855 | 1.57e-06** |
| TMEM67 | -0.880 | 3.06e-07** |
| SPTA1 | -0.915 | 1.66e-08** |

Supplementary Table 3. 68 Variants driving ClinVar correlation change. Annotation information and and associated diseases for the 68 Variants that drive the correlation between PAVs in ClinVar and African Ancestry to switch from positive to negative between March and April 2014.

| Chromosome | Position | ClinVar Accession ID | CLNDBN: Variant Disease Name Field |
|---|---|---|---|
| 1 | 11106666 | RCV000005520.1 | MASP2_deficiency |
| 1 | 21546501 | RCV000009704.3 | Hirschsprung_disease,cardiac_defects,and_autonomic_dysfunction |
| 1 | 45797228 | RCV000005614.6\|RCV000005615.2\|RCV000079501.3\|RCV000115748.4\|RCV000121598.1\|RCV000144637.1 | MYH-associated_polyposis\|Endometrial_carcinoma\|not_provided\|Hereditary_cancer-predisposing_syndrome\|not_specified\|Carcinoma_of_colon |
| 1 | 45799121 | RCV000119223.3\|RCV000126890.3,RCV000005617.3\|RCV000163049.1 | MYH-associated_polyposis\|Hereditary_cancer-predisposing_syndrome,MYH-associated_polyposis\|Hereditary_cancer-predisposing_syndrome |
| 1 | 63872032 | RCV000023375.1\|RCV000081558.5 | Congenital_disorder_of_glycosylation_type_1C\|not_specified |
| 1 | 94473287 | RCV000008346.1\|RCV000085773.3 | Stargardt's_disease\|not_provided |
| 1 | 94505604 | RCV000008361.1\|RCV000085583.1 | Cone-rod_dystrophy_3\|not_provided |
| 1 | 115231254 | RCV000077975.2 | not_provided |
| 1 | 172627498 | NA-not_in_'Current'_Clinvar_Version | NA-not_in_'Current'_Clinvar_Version |

| Chromosome | Position | ClinVar Accession ID | CLNDBN: Variant Disease Name Field |
|---|---|---|---|
| 1 | 182555149 | RCV000013878.23 | Prostate_cancer,hereditary,1 |
| 2 | 10188123 | RCV000006872.1 | Maturity-onset_diabetes_of_the_young, type_7 |
| 2 | 63131731 | RCV000001429.1 | Prostate_cancer,hereditary,12 |
| 2 | 136608646 | RCV000008124.1 | Lactase_persistence |
| 2 | 167129256 | RCV000023304.1\|RCV000080039.5 | Small_fiber_neuropathy\|not_specified |
| 2 | 167133540 | RCV000023304.1\|RCV000080038.5 | Small_fiber_neuropathy\|not_specified |
| 2 | 190925077 | RCV000055914.1 | Muscle_hypertrophy |
| 3 | 12393125 | RCV000118044.2 | not_specified |
| 3 | 165547569 | RCV000014116.23\|RCV000014117.16\|RCV000014118.23\|RCV000014119.23 | Bche,fluoride_2\|BCHE,FLUORIDE-RESISTANT_II\|CHE*390V\|BCHE*390V |
| 3 | 165548529 | RCV000014102.23\|RCV000014103.16 | Postanesthetic_apnea\|BCHE, dibucaine-resistant_i |
| 4 | 5755524 | RCV000005670.2 | Chondroectodermal_dysplasia |
| 4 | 187158034 | RCV000012817.23 | Prekallikrein_deficiency |
| 5 | 35072712 | RCV000074480.10 | Multiple_fibroadenomas_of_the_breast |
| 5 | 110454719 | RCV000001647.3 | Glaucoma_1,open_angle,G |

| Chromosome | Position | ClinVar Accession ID | CLNDBN: Variant Disease Name Field |
|---|---|---|---|
| 5 | 172662014 | RCV000009572.2\|RCV000009573.2\|RCV000023017.2\|RCV000023018.4\|RCV000023019.2\|RCV000030339.1\|RCV000037968.2\|RCV000146755.1 | Tetralogy_of_Fallot\|Hypothyroidism,congenital,nongoitrous,5\|Interrupted_aortic_arch\|Truncus_arteriosus\|Hypoplastic_left_heart_syndrome_2\|Congenital_heart_disease\|not_specified\|Malformation_of_the_heart_and_great_vessels |
| 6 | 18139228 | RCV000013559.22\|RCV000013561.16 | Thiopurine_methyltransferase_deficiency\|Thiopurine_methyltransferase_deficiency |
| 6 | 29080004 | RCV000033138.1 | C3hex,ability_to_smell |
| 6 | 29080344 | RCV000033139.1 | C3hex,ability_to_smell |
| 6 | 31910938 | RCV000012914.3 | Age-related_macular_degeneration_14 |
| 6 | 32007887 | RCV000055820.1,RCV000012934.2\|RCV000012935.1\|RCV000012936.1 | 21-hydroxylase_deficiency,21-hydroxylase_deficiency\|Adenoma,cortisol-producing\|Carcinoma,adrenocortical,androgen-secreting |
| 6 | 32008198 | RCV000012951.2 | 21-hydroxylase_deficiency |
| 7 | 99382096 | RCV000018417.2\|RCV000018418.23 | CYP3A4_PROMOTER_POLYMORPHISM\|Cyp3a4-v |

| Chromosome | Position | ClinVar Accession ID | CLNDBN: Variant Disease Name Field |
|---|---|---|---|
| 7 | 122635173 | RCV000005659.1\|RCV000005660.1 | Beta-glycopyranoside_tasting\|Alcohol_dependence,susceptibility_to |
| 7 | 142640113 | RCV000024078.1 | KEL6_ANTIGEN |
| 7 | 150878511 | RCV000043658.2 | Glaucoma_1,open_angle,F |
| 7 | 157160110 | RCV000024241.1,RCV000024240.1 | Limb-girdle_muscular_dystrophy,type_1E,Limb-girdle_muscular_dystrophy,type_1E |
| 9 | 6589230 | RCV000012765.16 | Non-ketotic_hyperglycinemia |
| 9 | 135781205 | RCV000005405.1\|RCV000042078.2\|RCV000118691.2\|RCV000125629.1\|RCV000163265.1 | Tuberous_sclerosis_1\|Tuberous_sclerosis_syndrome\|not_specified\|not_provided\|Hereditary_cancer-predisposing_syndrome |
| 10 | 51549496 | RCV000015312.24 | Prostate_cancer,hereditary,13 |
| 10 | 54531226 | RCV000015425.20 | Mannose-binding_protein_deficiency |
| 10 | 135348544 | RCV000018384.26 | CYP2E1*6_ALLELE |
| 11 | 27680107 | RCV000019266.26 | Congenital_central_hypoventilation |

| Chromosome | Position | ClinVar Accession ID | CLNDBN: Variant Disease Name Field |
|---|---|---|---|
| 11 | 46761055 | RCV000014237.17\|RCV000014238.1\|RCV000022729.1 | Thrombophilia\|Ischemic_stroke,susceptibility_to\|Pregnancy_loss,recurrent,susceptibility_to,2 |
| 11 | 69462910 | RCV000014762.3\|RCV000083293.3\|RCV000087019.3 | Colorectal_cancer,susceptibility_to\|Multiple_myeloma,translocation_11\x2c14_type\|VON_HIPPEL-LINDAU_SYNDROME,MODIFIER_OF |
| 12 | 6925407 | RCV000022781.22 | Okt4_epitope_deficiency |
| 12 | 121416650 | RCV000016074.1\|RCV000016075.25\|RCV000117233.3\|RCV000125370.1 | Insulin_resistance,susceptibility_to\|Serum_hdl_cholesterol_level,modifier_of\|not_specified\|not_provided |
| 14 | 75514138 | RCV000005900.1\|RCV000005901.1 | Endometrial_carcinoma\|Hereditary_nonpolyposis_colorectal_cancer_type_7 |
| 14 | 94847415 | RCV000019555.1\|RCV000019556.26,RCV000019553.1\|RCV000019554.26\|RCV000151834.1 | PI_M1-ALA213\|PI,M1V,PI_M1-ALA213\|PI,M1A\|not_specified |
| 15 | 28230318 | RCV000001014.2 | Skin/hair/eye_pigmentation,variation_in,1 |
| 15 | 28365618 | RCV000005011.2 | Skin/hair/eye_pigmentation,variation_in,1 |
| 16 | 3293403 | RCV000083740.1 | Familial_Mediterranean_fever |

| Chromosome | Position | ClinVar Accession ID | CLNDBN: Variant Disease Name Field |
|---|---|---|---|
| 16 | 16251599 | RCV000006948.1\|RCV000132640.1 | Pseudoxanthoma_elasticum\|not_provided |
| 16 | 48258198 | RCV000003737.3\|RCV000003738.2\|RCV000003739.2 | Apocrine_gland_secretion,variation_in\|Axillary_odor\|Colostrum_secretion |
| 17 | 3550800 | RCV000004700.2 | Cystinosis,atypical_nephropathic |
| 17 | 8790433 | RCV000041972.2 | Ataxia-oculomotor_apraxia_3 |
| 17 | 12899902 | RCV000005359.1 | Prostate_cancer,hereditary,2 |
| 17 | 16852187 | RCV000005623.1\|RCV000005624.1 | Common_variable_immunodeficiency_2\|Immunoglobulin_A_deficiency_2 |
| 19 | 7125518 | RCV000015822.26\|RCV000117280.1 | Diabetes_mellitus_type_2\|Pineal_hyperplasia_AND_diabetes_mellitus_syndrome |
| 19 | 41858921 | RCV000013360.25\|RCV000013361.2\|RCV000032141.1 | Cystic_fibrosis\|Breast_cancer,invasive,susceptibility_to\|Diaphyseal_dysplasia |
| 19 | 51323676 | RCV000015766.25 | Kallikrein,decreased_urinary_activity_of |
| 20 | 3193893 | RCV000015868.24 | Inosine_triphosphatase_deficiency |

| Chromosome | Position | ClinVar Accession ID | CLNDBN: Variant Disease Name Field |
|---|---|---|---|
| 21 | 44483184 | RCV000000141.3\|RCV000000142.3\|RCV000078111.3 | Homocystinuria,pyridoxine-responsive\|HYPERHOMOCYSTEINEMIA,THROMBOTIC,CBS-RELATED\|not_provided |
| 22 | 42524947 | RCV000018385.22 | Debrisoquine,poor_metabolism_of |
| 22 | 42526694 | RCV000018389.22 | Debrisoquine,poor_metabolism_of |
| X | 8536293 | RCV000010696.1 | Kallmann_syndrome_1 |
| X | 31496398 | RCV000012020.16\|RCV000080812.3\|RCV000124711.1 | Becker_muscular_dystrophy\|not_specified\|not_provided |
| X | 31496426 | RCV000012019.16\|RCV000080811.3\|RCV000124710.1 | Duchenne_muscular_dystrophy\|not_specified\|not_provided |
| X | 153763492 | RCV000011073.3\|RCV000011075.6\|RCV000011076.4\|RCV000011077.4\|RCV000011078.4\|RCV000011079.4\|RCV000011109.1\|RCV000079405.3 | G6PD_A+\|Glucose_6_phosphate_dehydrogenase_deficiency\|G6PD_BETICA\|G6PD_CASTILLA\|G6PD_DISTRITO_FEDERAL\|G6PD_TEPIC\|G6PD_SANTAMARIA\|not_provided |

| Chromosome | Position | ClinVar Accession ID | CLNDBN: Variant Disease Name Field |
|---|---|---|---|
| X | 153764217 | RCV000011075.6\|RCV000011076.4\|RCV000011077.4\|RCV000011078.4\|RCV000011079.4\|RCV000011157.2\|RCV000079404.3 | Glucose_6_phosphate_dehydrogenase_deficiency\|G6PD_BETICA\|G6PD_CASTILLA\|G6PD_DISTRITO_FEDERAL\|G6PD_TEPIC\|G6PD_ASAHI\|Anemia,nonspherocytic_hemolytic,due_to_G6PD_deficiency |

Supplementary Table 4. Thresholds for calling deleterious variants with *in silico* predictors. *In silico* prediction thresholds that were used in applying the deleteriousness filter are shown. A variant had to be in the top ten percent of possible deleteriousness scores for a predictor in order to be considered deleterious by that prediction method. Two different prediction methods, out of eleven, were required to pass the deleteriousness filter.

| *in silico* predictor from ANNOVAR | Threshold (10th percentile) |
|---|---|
| LR score[7] | ≥0.695 |
| RadialSVM score[7] | ≥0.425 |
| MutationAssessor score[8] | ≥3.085 |
| phyloP 46way placental[9] | ≥2.648 |
| SiPhy 29way logOdds[10] | ≥18.213 |
| Polyphen2 HVAR score[11] | ≥0.999 |
| GERP++ RS[12] | ≥5.73 |
| CADD Phred Score[2] | ≥20 |
| LRT score[13] | "="0 |
| SIFT score[14] | “=“0 |
| FATHMM score[15] | ≤-2.45 |

Supplementary Table 5. Classifier profile for deleterious PAVs and deleterious NAVs. For each of 11 *in silico* prediction methods, counts are shown for the total number of deleterious PAVs and deleterious NAVs called deleterious by that predictor. The percentage of total deleterious calls for PAVs and NAVs made up by each predictor is also shown. The column labeled "Percentile Difference" shows the difference between deleterious PAVs and deleterious NAVs as a percentage of total deleteriousness hits made by each predictor. The larger the percentage of difference, the larger the percentage of total deleteriousness hits that was called by that predictor in PAVs compared to NAVs. Conversely, the smaller the percentage of difference, the larger the percentage of total deleteriousness hits that was called by that predictor in NAVs compared to PAVs. Percent differences ≤ -1% are seen in conservation dominant algorithms, signifying that conservation algorithms make up a higher percentage of deleterious calls amongst NAVs. Percentage differences ≥1% are seen in machine learning dominant algorithms, representing that machine learning algorithms make up a higher percentage of deleterious calls amongst PAVs. These differences may explain why the application of the deleterious prediction filter seems to reduce the positive correlation in PAVs by a much greater amount than in NAVs.

| **Predictor** | **Del PAV Count** | **Del PAV %** | **Del NAV Count** | **Del NAV %** | **% Difference** | **Train with Clinical Data** |
|---|---|---|---|---|---|---|
| FATHMM[15] | 3442 | 0.1306 | 3682 | 0.0762 | 0.0543 | yes |
| SIFT[14] | 2278 | 0.0864 | 5947 | 0.1232 | -0.0367 | no |
| LRT[13] | 5229 | 0.1984 | 11268 | 0.2333 | -0.0350 | no |
| LR[7] | 2665 | 0.1011 | 3356 | 0.0695 | 0.0316 | yes |
| SiPhy 29way logOdds[10] | 1492 | 0.0566 | 3452 | 0.0715 | -0.0149 | no |
| CADD[2] | 3308 | 0.1255 | 6847 | 0.1418 | -0.0163 | no |
| phyloP 46way placental[9] | 1467 | 0.0556 | 3178 | 0.0658 | -0.0102 | no |
| GERP++ RS[12] | 1138 | 0.0432 | 2088 | 0.0432 | -7.15E-05 | no |
| Polyphen2 HVAR[11] | 1510 | 0.0573 | 3060 | 0.0634 | -0.0061 | yes |
| RadialSVM[7] | 2508 | 0.0951 | 2865 | 0.0593 | 0.0358 | yes |
| MutationAssessor[8] | 1325 | 0.0503 | 2546 | 0.0527 | -0.0025 | no |

Supplementary Table 6. Cost survey of custom single variant clinical validation. Cost and information pertaining to single variant validation options at different institutions are shown. As can be seen, dollar amounts are between $240-$920 per variant

| **Lab** | **Test** | **Price** | **Website** |
|---|---|---|---|
| Medical College of Wisconsin- Developmental and Neurogenetics Sequencing Laboratory | Custom Clinical Sanger Sequencing | $275.00 | http://www.hmgc.mcw.edu/clinical/tests/CCS.htm |
| Cincinnati Children's Hospital Medical Center, Molecular Genetics Laboratory | Custom Gene Sequencing | $684.89 | http://www.cincinnatichildrens.org/molecular-genetics/ |
| Emory University School of Medicine, Emory Molecular Genetics Laboratory | Familial Mutation Testing: Targeted Sequencing | $350.00 | http://www.geneticslab.emory.edu/ |
| Baylor College of Medicine, Medical Genetics Laboratories | Custom Proband Sequence Analysis (1 amp) | $550 (insurance), $920 (private payment) | http://www.bcm.edu/geneticlabs/ |
| UCLA | Custom Proband Sequencing | $240.00 | http://www.ncbi.nlm.nih.gov/pubmed/24406459 |
| University of Chicago Genetic Services Lab | Custom mutation analysis of Proband | $540.00 | http://dnatesting.uchicago.edu/sites/default/files/01CustomMutAnalysis_5.pdf |
| University of Chicago Genetic Services Lab | Custom mutation analysis of additional family members | $390.00 | http://dnatesting.uchicago.edu/sites/default/files/01CustomMutAnalysis_5.pdf |

Supplementary Note 1

In order to explore the change over time in correlation of ancestry with PAVs from ClinVar[1], we analyze the list of variants that differed between March and April 2014, which represented the months with the largest correlation difference. For each of these variants, we filter on allele frequencies, protein function, *in silico* predictions of deleteriousness, and then calculate the correlation between African ancestry and the total number of ClinVar[1] PAVs at this site in all individuals. This correlation is weighted as described in the main text, and is calculated in the same way as the overall correlations with ancestry for each ClinVar[1] data release time point. Starting with the complete ClinVar[1] variant data from April 2014, we selectively include and exclude any of these variants that differ between March and April 2014 depending on their correlation coefficients and significance. Variants from the March release that are missing from the April release and had significant positive or negative correlations ($p \leq 0.05$), are added to the April variant data, while variants from the April release that are not in the March release and had significant positive or negative correlations ($p \leq 0.05$) are subtracted from the April variant data.

After adding or subtracting, we identify a total of 68 variants (see Supplementary Table 4) that largely recapitulate the correlation differences. These 68 SNVs come from every chromosome, and do not have a particularly different distribution of database origin or disease association. When selectively considering these 68 SNVs, we are able to recapitulate 94.65% of March's correlation (r=0.733), thus altering April's correlation coefficient (r) from -0.683 to 0.658. Since April 2014, there has been a steady increase

in the number of overall pathogenic variants in the ClinVar[1] database (Figure 2, black line), and with it, a rebound in the correlation of ancestry with total number of PAVs from ClinVar[1] per individual. Interestingly, the general reintroduction into ClinVar[1] of these 68 SNVs corresponds to when the correlation rebounded in the July 2, 2014 version and all subsequent versions. Since the biases for African Americans, and likely non-Europeans in general, clearly change as this database evolves, and this effects interpretation of variation, it is important for the medical genetics and precision medicine communities to regularly evaluate how to best use ClinVar[1], particularly for minority patients.

## Supplementary Discussion

### Asthma Focus In The CAAPA Dataset

The CAAPA cohort consists of samples collected for investigation into the genetics of asthma. To verify our assumption that the ascertainment of individuals with asthma should not effect our results or enrich for pathogenic, deleterious, and/or truly causal variants, we used our per gene analysis framework to demonstrate that genes implicated in asthma have no meaningful effect on our results and conclusions. After calculating ancestry-based bias in each gene, we looked at the subset of genes with the most bias, including genes with both meaningfully positive and negative correlations between African-ancestry and pathogenic variant counts per gene. Using subsets of the most bias genes (even before multiple testing correction), we found no evidence at all for enrichment of any disease networks or pathways, as annotated by the gene ontology consortium database (GO), as well as by curated Mendelian, recessive, dominant, and X-linked genes. Furthermore, we found no evidence in highly biased genes for

enrichment of GWAS catalogue genes, which should contain any genes that were the most significant hits in any GWAS, including those looking at associations with asthma. Finally, the most significantly biased genes after multiple testing (~10) have not been implicated in asthma.

Ancestry specific genomic data and databases

One might ask what the increasing numbers of whole African-ancestry genomes being deposited into public resources (through NHBLI and NHGRI etc) may do to the biases we report here, and whether such action might cause these biases to disappear. While such increased sequencing of whole African-ancestry genomes is surely a step in the right direction, one serious limitation to the disappearing of the biases we report is that most of the current and upcoming African-ancestry genome sequencing is not being done on cohorts that have the necessary and robust phenotype data that comparable studies of predominantly European-ancestry individuals use to populate databases such as ClinVar (i.e. in annotating variants as pathogenic etc). Instead, these African-centric studies are more focused on the complex disease genetics that underlie medical illnesses in foundational areas such as cardiology, pulmonology, and psychiatry. In addition, even if this phenotype data did exist, we still believe it would take significant time for the amount of African data in the databases to “catch up” to the dominant amount of European data currently populating these databases. Finally, if the databases were to theoretically become predominantly and disproportionately populated with data specific to African populations, our results suggest that other ancestry related biases might develop for non-African ancestry populations. Therefore, the genetics

community must be aware of the importance of accounting for population specificities, particularly when using databases to prioritize variants in the context of precision genomic medicine.

## Supplementary Methods

### Per gene analysis

To explore the correlation between PAVs in ClinVar[1] and African ancestry further, we conduct a similar correlation analysis on a per gene basis. By counting up the total number of PAVs in each gene for each person, we run a weighted correlation analysis as described above on each of 24,043 human genes as annotated in UCSC's hg19 RefGene list. After multiple testing correction, only 3 genes have significant correlations (Supplementary Table 2). Since many of the genes had very small total numbers of PAVs, even across all individuals, we rerun the correlation analysis after excluding all genes with less than 5 total PAVs across all individuals. This leaves a total of 645 genes, and by cutting away the multitude of underpowered genes with low counts, we identify 10 genes with significant correlations after multiple testing correction (Supplementary Table 2). For both analyses, follow-up is qualitatively the same, and so we describe in the main text approaches and results for the larger full gene analysis. The fact that our filtering of low count genes does little to quantitatively change our follow-up analysis, even after the removal of over 97% of genes, provides support that raising the minimum number of PAVs per gene further would do little to increase our power or improve our analysis.

### Gene enrichment analyses

After calculating correlation value per gene, we make a list of 74 genes that have a significant positive association before multiple testing correction and another list of 198 genes that have a significant negative correlation before multiple testing correction. Using additional gene lists compiled from OMIM[3], ClinVar[1], and HGMD[4], and the GWAS catalogue[5], we find no significant enrichment for Mendelian, dominant, recessive, X-linked or GWAS catalogue genes amongst positive and negative correlation genes (Pearson's Chi-squared test and Wilcoxon rank sum test). Our lists overlap and contain 2050 mendelian genes, 670 dominant genes, 1050 recessive genes, 491 X-linked genes, and 5045 GWAS catalogue genes. GWAS catalogue genes are defined as genes that contain at least one variant that was a top genome wide hit in a GWAS study of a complex trait.[5] We also test whether Mendelian, dominant, recessive, X-linked or GWAS catalogue genes have different correlation values than genes outside of these categories, but results are non-significant in each of these cases. As we found no evidence of an enrichment of highly biased genes in any of the annotated mendelian, recessive, or dominant gene categories, as would be expected if a model based on dominance was particularly relevant to our results, we feel that the additive approach we have taken is best. Additionally, since our goal in assessing the variants present in each individual is to build up population level evidence, it is important to consider each allele independently in assessing the population wide evidence of the likelihood that a variant is casual. Using the GORILLA program[6], we tested our significant positive and negative correlation gene lists for enrichment of GO terms, but results were unremarkable for all tests, especially at genome-wide significance levels.

## Supplementary References


1. Landrum, M.J. *et al.* ClinVar: public archive of relationships among sequence variation and human phenotype. *Nucleic Acids Res* **42**, D980-5 (2014).
2. Kircher, M. *et al.* A general framework for estimating the relative pathogenicity of human genetic variants. *Nature genetics* **46**, 310-315 (2014).
3. Amberger, J.S., Bocchini, C.A., Schiettecatte, F., Scott, A.F. & Hamosh, A. OMIM.org: Online Mendelian Inheritance in Man (OMIM(R)), an online catalog of human genes and genetic disorders. *Nucleic Acids Res* **43**, D789-98 (2015).
4. Stenson, P.D. *et al.* The Human Gene Mutation Database: building a comprehensive mutation repository for clinical and molecular genetics, diagnostic testing and personalized genomic medicine. *Hum Genet* **133**, 1-9 (2014).
5. Hindorff, L.A., Junkins, H.A., Hall, P., Mehta, J. & Manolio, T. A catalog of published genome-wide association studies. *National Human Genome Research Institute* (2011).
6. Eden, E., Navon, R., Steinfeld, I., Lipson, D. & Yakhini, Z. GOrilla: a tool for discovery and visualization of enriched GO terms in ranked gene lists. *BMC Bioinformatics* **10**, 48 (2009).
7. Dong, C. *et al.* Comparison and integration of deleteriousness prediction methods for nonsynonymous SNVs in whole exome sequencing studies. *Human molecular genetics* **24**, 2125-2137 (2015).
8. Reva, B., Antipin, Y. & Sander, C. Predicting the functional impact of protein mutations: application to cancer genomics. *Nucleic acids research*, gkr407 (2011).
9. Siepel, A., Pollard, K.S. & Haussler, D. New methods for detecting lineage-specific selection. in *Research in Computational Molecular Biology* 190-205 (Springer, 2006).
10. Garber, M. *et al.* Identifying novel constrained elements by exploiting biased substitution patterns. *Bioinformatics* **25**, i54-i62 (2009).
11. Adzhubei, I.A. *et al.* A method and server for predicting damaging missense mutations. *Nature methods* **7**, 248-249 (2010).
12. Davydov, E.V. *et al.* Identifying a high fraction of the human genome to be under selective constraint using GERP++. *PLoS Comput Biol* **6**, e1001025 (2010).
13. Chun, S. & Fay, J.C. Identification of deleterious mutations within three human genomes. *Genome research* **19**, 1553-1561 (2009).
14. Kumar, P., Henikoff, S. & Ng, P.C. Predicting the effects of coding non-synonymous variants on protein function using the SIFT algorithm. *Nature protocols* **4**, 1073-1081 (2009).
15. Shihab, H.A., Gough, J., Cooper, D.N., Day, I.N. & Gaunt, T.R. Predicting the functional consequences of cancer-associated amino acid substitutions. *Bioinformatics*, btt182 (2013).
